# Application of an Open‐Source Modular Framework for Pharmacokinetic, Pharmacodynamic, and Safety Simulations to Anti‐Tuberculosis Drugs

**DOI:** 10.1002/psp4.70347

**Published:** 2026-10-06

**Authors:** Marco Siccardi, Nina Nauwelaerts, Raphaëlle Lesage, Diane Lefaudeux, Vanessa Baier, Venetia Karamitsou, Cleo Demeester, Miao‐Chan Huang, Alexander Kulesza, Stephan Schaller

**Affiliations:** ^1^ ESQlabs GmbH Saterland Germany; ^2^ University of Liverpool Liverpool UK

## Abstract

Tuberculosis (TB) treatment requires long‐term multidrug regimens where dosing, efficacy, and safety optimization remain challenging. This tutorial establishes an integrated, open‐source, modular framework connecting high‐throughput PBPK (HT‐PBPK) simulations, mechanistic PBPK models, pharmacodynamic endpoints, and safety predictions for anti‐TB drugs. Using the Open Systems Pharmacology Suite and ESQhtpbpk (R package), physicochemical parameters were obtained from literature, with QSAR‐predicted values supplementing missing experimental data to generate generic PBPK models. Mechanistic models were parameterized for moxidectin, isoniazid, moxifloxacin, bedaquiline, and rifampicin. A quantitative TB disease model of host‐pathogen dynamics and PK‐PD interactions was integrated to assess efficacy. A cardiac electrophysiology model incorporating ion channel inhibition data was coupled with PBPK predictions for moxifloxacin and bedaquiline. The HT‐PBPK framework automated virtual studies across compounds and dosing regimens. Mechanistic models reproduced systemic and local disposition within two‐fold of the observed data. The TB disease model predicted treatment response across exposure scenarios. The cardiac module captured the dose‐QTc relationship for moxifloxacin and identified metabolite‐driven QTc prolongation with bedaquiline, consistent with clinical observations. This open‐source, modular framework spans compound screening, mechanistic PK, efficacy, and cardiotoxicity, providing a reproducible tool to support TB drug development, guide dose selection, and inform regulatory decisions.

## 1 Introduction

Tuberculosis (TB) remains one of the leading causes of infectious disease mortality worldwide. The World Health Organization estimated over 10.8 million new TB cases in 2023, with more than 1.25 million deaths, including among people living with HIV co‐infection [1]. Treatment requires prolonged multidrug regimens lasting months to years, and optimizing dosing, efficacy, and safety across heterogeneous patient populations, including those with comorbidities, drug‐resistant disease, and limited access to clinical infrastructure, represents a substantial and ongoing challenge. The long duration of treatment, complex drug combinations, and the risk of acquired resistance mean that rational regimen design cannot rely on empirical approaches alone.

Mechanistic modeling and simulation provide a structured approach to connect pharmacokinetic, pharmacodynamic, and safety data within a unified quantitative framework. Physiologically based pharmacokinetic (PBPK) modeling predicts drug disposition across tissues and populations using physiological parameters, enabling translation from preclinical to clinical settings and across special populations including those with organ impairment or comedication [2]. When coupled with pharmacodynamic models describing bacterial killing, lesion penetration, and resistance dynamics, PBPK can support dose selection, regimen optimization, and identification of high‐risk scenarios that warrant experimental investigation. For anti‐TB drugs, which must reach intracellular and granulomatous sites and are frequently used in combinations that carry cardiac or hepatic safety liabilities, integrated quantitative frameworks are of particular value [3].

Despite the growing application of modeling in drug development, most PBPK and quantitative systems pharmacology (QSP) tools remain proprietary, limiting their accessibility in resource‐constrained settings where the TB burden is highest [4]. Models are frequently developed in isolation, a PBPK model in one software environment, a pharmacodynamic model in another, and a safety module in a third, making integration across the development continuum difficult and reducing reproducibility. The absence of openly available, documented, and interoperable modeling resources represents a meaningful gap, particularly for global health applications where research infrastructure and regulatory capacity may be limited.

This tutorial describes an integrated, open‐source, modular framework for anti‐TB drug modeling, built on the Open Systems Pharmacology (OSP) Suite [4] and the R environment, and distributed through public GitHub repositories. The framework combines four connected components: a high‐throughput PBPK (HT‐PBPK) pipeline for rapid compound screening across large libraries using physicochemical and in silico inputs; mechanistic PBPK models for priority TB drugs that reproduce observed clinical pharmacokinetics across species and populations; QSP modules including (1) a cardiac electrophysiology model that translates drug exposure into ion‐channel inhibition and the resulting cardiomyocyte action potential delay, and (2) data‐driven QT‐prolongation prediction models; and a mechanistic TB disease model that links drug concentrations in lung interstitium and granulomatous lesions to bacterial killing, immune dynamics, and long‐term treatment outcomes. Each component can be used independently, but their integrated operation creates a coherent system that can accompany a compound from early discovery through translational and clinical assessment.

The aim of this tutorial is to demonstrate how these components can be applied, independently and in combination, to support TB drug development decisions. Using five representative anti‐TB drugs, rifampicin, isoniazid, moxifloxacin, bedaquiline, and moxidectin, we illustrate the workflow from automated exposure screening to mechanistic PK modeling, pharmacodynamic integration, and cardiac safety assessment. All models, code, and documentation are openly accessible through the repositories described throughout this paper, enabling reproduction, validation, and extension of the framework by the broader research and regulatory community, including groups working in settings where proprietary tools are not available.

## 2 An Integrated Approach to Anti‐TB Drug Modeling

Tuberculosis drug development faces structural and scientific challenges. Datasets in the field have traditionally been fragmented, with in vitro potency measurements, in vivo pharmacokinetic profiles, and clinical outcomes generated at different stages and across different experimental systems, without a unifying framework to connect molecular properties to human treatment outcomes. Each dataset provides partial insight but lacks the context to answer the questions most relevant to development decisions: whether a given exposure level is likely to achieve sterilization in the relevant bacterial niches, how cardiac risk changes across dose scenarios and patient characteristics, and how resistance emergence responds to differences in regimen composition. An integrated modeling approach addresses this by connecting data and predictions through a layered quantitative structure rather than treating each question as an isolated analytical exercise.

The framework described in this tutorial operates through three connected simulation approaches. The first is a high‐throughput PBPK layer that uses physicochemical data and quantitative structure–activity relationship predictions to generate rapid estimates of systemic and tissue exposure for large compound sets, enabling early prioritization before detailed in vivo or clinical data are available. The second is a mechanistic PBPK layer that builds on the most promising compounds, incorporating metabolic pathways, transport processes, and tissue‐specific properties to produce physiologically realistic exposure predictions across species and populations. The third is a quantitative systems pharmacology layer that integrates PBPK‐predicted concentrations into mechanistic modules describing safety and efficacy, specifically cardiac electrophysiology and TB bacterial dynamics. Each layer informs the next, ensuring that early screening predictions can evolve into detailed mechanistic analyses as evidence accumulates and compound characterization deepens.

The clinical rationale for an integrated framework is reinforced by the pharmacological characteristics of the TB drug class. Current regimens combine four or more drugs with distinct mechanisms of action, tissue penetration profiles, and metabolic profiles. Rifampicin is a potent inducer of cytochrome P450 enzymes and drug transporters, creating clinically significant drug–drug interaction scenarios for co‐administered medicines including antiretrovirals and oral contraceptives [5]. Bedaquiline has a long effective half‐life, accumulates in peripheral tissues, and prolongs the QT interval through its active M2 metabolite. Moxifloxacin contributes to both antibacterial activity and an independent cardiac liability. Isoniazid exhibits a bimodal pharmacokinetic profile driven by N‐acetyltransferase 2 genetic polymorphism, with fast and slow acetylators reaching substantially different systemic exposures at the same nominal dose. These overlapping properties mean that the questions most relevant to regimen optimization cannot be addressed by pharmacokinetic analysis alone: they require pharmacodynamic context and prospective safety evaluation to generate genuinely useful predictions.

All components of the framework are implemented using open‐source tools to maximize accessibility, reproducibility, and long‐term utility. The OSP Suite, including PK‐Sim and MoBi, and the R statistical computing environment are both freely available and supported by active developer and regulatory communities. Models and code are distributed through public repositories, with structured documentation that describes model assumptions, parameterization choices, and simulation workflows in sufficient detail to allow independent reproduction. This approach is aligned with the transparency principles promoted by international regulatory guidance on PBPK reporting and with the goals of open science more broadly. For researchers and developers working in settings where commercial modeling platforms are not available, this open architecture provides a practical entry point into model‐informed TB drug development without requiring proprietary software licenses or institutional subscriptions.

## 3 High‐Throughput Physiologically Based Pharmacokinetic Framework

### 3.1 Framework Design and Implementation

The increasing adoption of PBPK modeling in drug development has created a demand for scalable, automated workflows that efficiently simulate hundreds of compounds. Traditional PBPK model construction provides mechanistic depth but is resource‐intensive, requiring the assembly of compound‐specific data, manual model building, and individual verification for each molecule. These requirements place practical limits on throughput at early development stages, where decision‐making must span large compound sets with incomplete experimental data. A high‐throughput pipeline allows scaling the simulation of many compounds and many administration designs in an efficient way by integrating it with large databases and automating PBPK model generation and simulation. The ESQhtpbpk R package was developed to address this need by automating model generation, parameterization, and simulation for large compound libraries within the open‐source OSP ecosystem as an initial screening and prioritization tool. The framework is implemented entirely in R and eases the integration of in vitro and in silico physicochemical data to generate in vivo pharmacokinetic predictions while maintaining full transparency, version control, and reproducibility [6]. The complete package, documentation, and example workflows are freely available at https://github.com/esqLABS/ESQhtpbpk.

The pipeline leverages modular OSP components, specifically PK‐Sim and MoBi [4, 7], to build generic PBPK structures that automatically adapt to compound‐specific properties and study designs. The generic PBPK model structure can be automatically built for any species supported by PK‐Sim (human, rat, mouse, monkey, dog, …) using any of the partition coefficients (PK‐Sim Standard, Rodgers and Rowland, Poulin and Theil, Berezhkovskiy, Schmitt) and cellular permeability methods implemented (PK‐Sim Standard, Schmitt), and using any of the following biological processes (linear hepatic clearance, renal clearance, biliary clearance). Automatic model generation uses default OSP physiological templates, populated with compound descriptors such as logP, pKa, aqueous solubility, membrane permeability, and plasma protein binding. These inputs are derived from curated experimental databases or, where experimental values are unavailable, from quantitative structure–activity relationship tools that predict properties from molecular structure alone. Batch simulation across multiple compounds, species, and dosing regimens is executed through the ospsuite runsimulationBatches() function, enabling parallel processing of large compound libraries without manual intervention. The framework generates concentration‐time profiles for each study from which automated analysis of exposure metrics, including area under the concentration‐time curve (AUC), maximum observed concentration (Cmax), and time to maximum concentration (Tmax) can be derived, together with graphical summaries comparing predicted and observed profiles when reference data are available. Examples of both can be found in the documentation. The standardized workflow structure ensures reproducibility and scalability across hundreds of compounds and experimental designs while maintaining compatibility with the downstream mechanistic PBPK and quantitative systems pharmacology models described in subsequent sections of this tutorial.

A defining feature of the approach is its use of quantitative structure–activity relationship (QSAR)‐predicted inputs when experimental measurements are absent. Physicochemical properties, including partition coefficients, ionization constants, and aqueous solubility, can be estimated computationally from molecular structure using established predictive tools, providing the parameter set needed to instantiate a PBPK model without requiring dedicated wet laboratory work for each compound. This enables the application to compound classes at the discovery stage, where synthesizing large chemical libraries makes experimental pharmacokinetic profiling impractical for every molecule. The predicted inputs carry inherent uncertainty, which propagates into the resulting exposure estimates and must be interpreted accordingly. Thus, the application domain of the HT‐PBPK framework should be restricted to the applicability domain of the used QSARs and to the general applicability of the PBPK model. The ESQhtpbpk framework is therefore positioned as a screening and prioritization tool that identifies which compounds merit more detailed mechanistic analysis rather than as a source of definitive quantitative predictions for regulatory or clinical purposes. An assessment of the prediction accuracy of AUC and Cmax using data extracted from the ChEMBL database, as well as a small set of 12 antituberculosis drugs, has been conducted and is available as an example in the GitHub repository. A worked example applying the framework to anti‐TB compounds is available at https://esqlabs.github.io/ESQhtpbpk/articles/Example‐with‐anti‐TB‐drugs.html. An overview of the workflow of the high‐throughput PBPK framework can be found in the Figure S1.

To make the scientific basis of the approach explicit, its structure and parameters are summarized here. Each compound is represented by a whole‐body PBPK model generated from the standard OSP/PK‐Sim physiology4, in which the arterial and venous blood pools and the major organ compartments (including lung, heart, liver, kidney, gut, spleen, muscle, skin, fat, bone and the remaining tissue) are represented as well‐stirred compartments connected by the corresponding organ blood flows and cardiac output. Passive tissue distribution is described by tissue‐to‐plasma partition coefficients calculated from the compound physicochemistry using a standard OSP distribution model, and systemic elimination is parameterised from the available clearance information for each compound, or predicted where such data are absent. The compound‐specific input parameters required to instantiate this structure are molecular weight, lipophilicity (logP/logD), acid–base character (pKa and compound type), aqueous solubility, intestinal permeability, the fraction unbound in plasma and, where available, the blood‐to‐plasma ratio; these are taken from curated experimental databases when measured values exist and are otherwise predicted from molecular structure using QSAR tools. Study‐specific inputs comprise the administration route, dose, formulation and dosing schedule together with the species and population template. From these inputs the model predicts plasma and tissue concentration‐time profiles, from which the exposure metrics used for triage (AUC, Cmax and Tmax) are derived. A complete list of input parameters, their sources and the predicted outputs is provided in Table S1.

### 3.2 Application to Anti‐Tuberculosis Compounds

To evaluate the performance of the ESQhtpbpk framework under conditions directly relevant to TB drug development, the pipeline was applied to a dataset of 12 anti‐TB drugs spanning a range of chemical classes, physicochemical properties, and routes of administration, including intravenous and oral dosing, and multiple dosing regimens drawn from published clinical studies. Over 100 individual simulations were executed using literature‐based study designs and QSAR‐predicted physicochemical inputs. Model performance was evaluated by comparing predicted and observed in vivo pharmacokinetic data across all studies, using the predicted‐to‐observed ratio and fold error as primary performance metrics.

The evaluation demonstrated that 38%–42% of AUC and Cmax predictions fell within a two‐fold error of the observed values, and 60% of AUC predictions and 75% of Cmax predictions fell within a four‐fold error across all studies (Figure 1). Median predicted‐to‐observed ratios were 0.6‐fold for AUC and 1.3‐fold for Cmax. Systematic underestimation of AUC was observed for highly lipophilic compounds with low aqueous solubility and low unbound plasma fraction (Figure S2), an expected limitation of generic absorption and distribution estimates applied without compound‐specific calibration of partition parameters. These results are consistent with published benchmarks for generic PBPK performance using computational inputs across diverse compound libraries [6], and they confirm that the framework is suited to its intended role as a rapid triage and prioritization tool. The level of prediction accuracy achieved is sufficient to rank compounds by exposure, identify molecules with predicted tissue distribution characteristics favorable for TB drug development, and flag compounds with predicted safety margins that warrant further investigation before in vivo studies are initiated.

**FIGURE 1:**
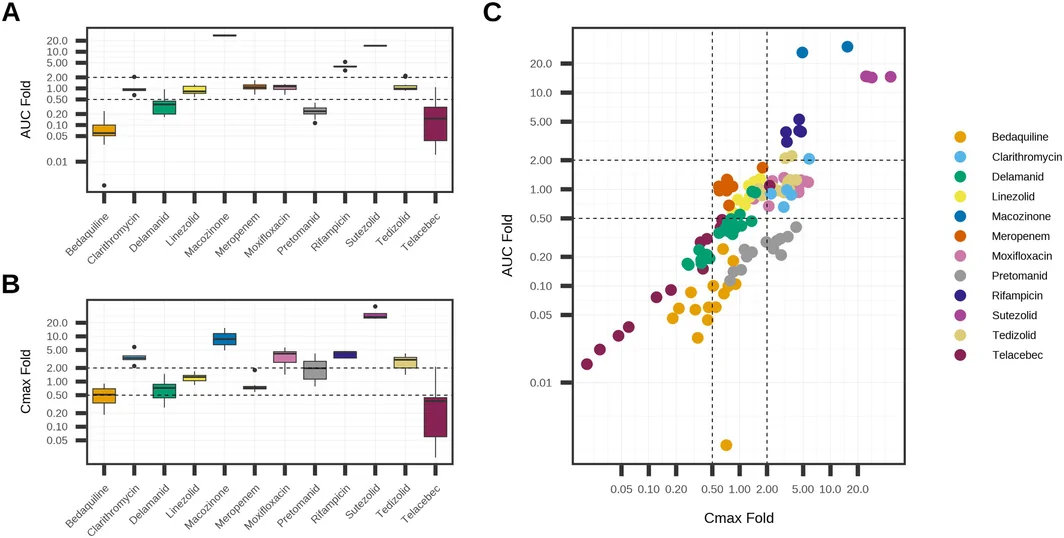
Predictive performance of the high‐throughput PBPK framework applied to anti‐tuberculosis compounds using QSAR‐derived physicochemical inputs. Box‐whisker plots depicting predicted versus observed AUC fold change (panel A) and Cmax fold change (panel B) are shown for 12 anti‐TB drugs across over 100 simulations. The boxes represent the interquartile range (IQR), with the horizontal lines inside the boxes representing the median. The individual study predictions are also shown with their AUC and Cmax fold values (panel C). In all graphs, the dashed lines indicate two‐fold error boundaries.

### 3.3 Role Within the Integrated Framework

Within the framework, HT‐PBPK is the first filter: a way to organize early compound information into exposure estimates before the effort of full mechanistic modeling is warranted. Rapid prioritization based on predicted tissue exposure relative to pharmacodynamic targets guides which compounds merit the more detailed analyses in subsequent sections. Future additions will include population variability modules and more direct linkage to the cardiac safety and disease model components.

The HT‐PBPK framework also serves an important role in supporting collaborative and resource‐limited research environments. Because the entire workflow runs within R and relies only on open‐source dependencies, it can be deployed on standard computing hardware without specialized infrastructure. Researchers in low‐ and middle‐income countries, where the TB burden is concentrated and access to commercial pharmacokinetic software is often constrained by licensing costs, can use the same modeling tools as well‐resourced academic or industry groups. The availability of the full workflow through a public repository with versioned releases further supports reproducibility and allows modelers to track how predictions change as the underlying physicochemical databases and QSAR tools are updated.

## 4 Mechanistic Physiologically Based Pharmacokinetic Models for Anti‐Tuberculosis Drugs

### 4.1 Scientific Context and Modeling Objectives

Mechanistic PBPK modeling provides the quantitative core of the integrated anti‐TB framework, translating compound‐specific properties and physiological parameters into predictions of drug disposition across tissues, populations, and time. While the HT‐PBPK pipeline is suited to early compound prioritization using generic model structures and computationally predicted inputs, mechanistic PBPK modeling is applied to compounds that have advanced beyond initial screening, using experimentally derived physicochemical data, metabolic parameters measured in relevant in vitro systems, and clinical pharmacokinetic observations to build models that accurately reproduce the full concentration‐time profile in plasma and target tissues. The mechanistic PBPK models form the link between in vitro and *in silico* data collected early in development and the downstream pharmacodynamic and safety analyses that require realistic, population‐representative exposure inputs for meaningful interpretation.

In the context of TB drug development, the target tissues are not the plasma. Drug activity against *Mycobacterium tuberculosis* depends on concentrations achieved in lung interstitial fluid, within the cytoplasm of alveolar macrophages, and in the anaerobic core of granulomatous lesions, each of which has distinct physiological properties affecting drug partitioning, membrane permeability, and local binding. Plasma pharmacokinetics can serve as a useful surrogate when the relationship between plasma and tissue exposure is well characterized and proportional, but it cannot substitute for mechanistic tissue models when this relationship is uncertain, compound‐specific, or modified by disease state. The mechanistic PBPK models developed in this framework are therefore parameterized to reproduce both systemic and tissue concentration profiles, providing the quantitative inputs needed to drive the TB disease model, which explicitly simulates drug effects in lung interstitial and lesion compartments.

### 4.2 Model Library and Predictive Performance

Mechanistic PBPK models have been developed and qualified for five priority anti‐TB drugs: rifampicin, isoniazid, moxifloxacin, bedaquiline, and moxidectin. All models are implemented in the OSP Suite and formatted for direct import and use in PK‐Sim and MoBi. They are hosted on public repositories with structured model files, evaluation reports documenting parameter sources, qualification datasets, and simulation scripts that enable transparent reuse and independent reproduction by other researchers. The models are interoperable with the downstream components of the framework and can be extended to incorporate additional dosing regimens, special populations, or drug–drug interaction scenarios without rebuilding from scratch. An overview of the used models and the quantitative model performance, measured as geometric mean fold error (GMFE), is presented in Table 1. The observed and simulated concentration‐time profiles are shown in Figure 2.

**TABLE 1:** Overview PBPK model availability and performance of several anti‐TB drugs.

Model	Open access	Applicability	Performance
Rifampicin	Rifampicin Model GitHub [8]	9 studies, IV and oral (300–600 mg); DDI qualified	GMFE = 1.28 AUC fold = 1.28
Bedaquiline	Bedaquiline Model GitHub [9]	3 oral studies (200–700 mg)	GMFE = 1.13 AUC fold = 1.14
Isoniazid	Isoniazid Model GitHub [10]	11 studies IV/oral (4.75–20 mg/kg; 300–900 mg); acetylator phenotypes	GMFE = 1.23 AUC fold = 1.30
Moxidectin	Moxidectin Model GitHub [11]	4 oral studies (3–36 mg) fed/fasted	GMFE = 1.38 AUC fold = 1.16
Moxifloxacin	Moxifloxacin Model GitHub [12]	6 studies IV/oral (60–600 mg)	GMFE = 1.28 AUC fold = 1.30

Abbreviations: AUC, area under the curve; DDI, Drug–drug interaction; GMFE, geometric mean fold error; IV, intravenous.

**FIGURE 2:**
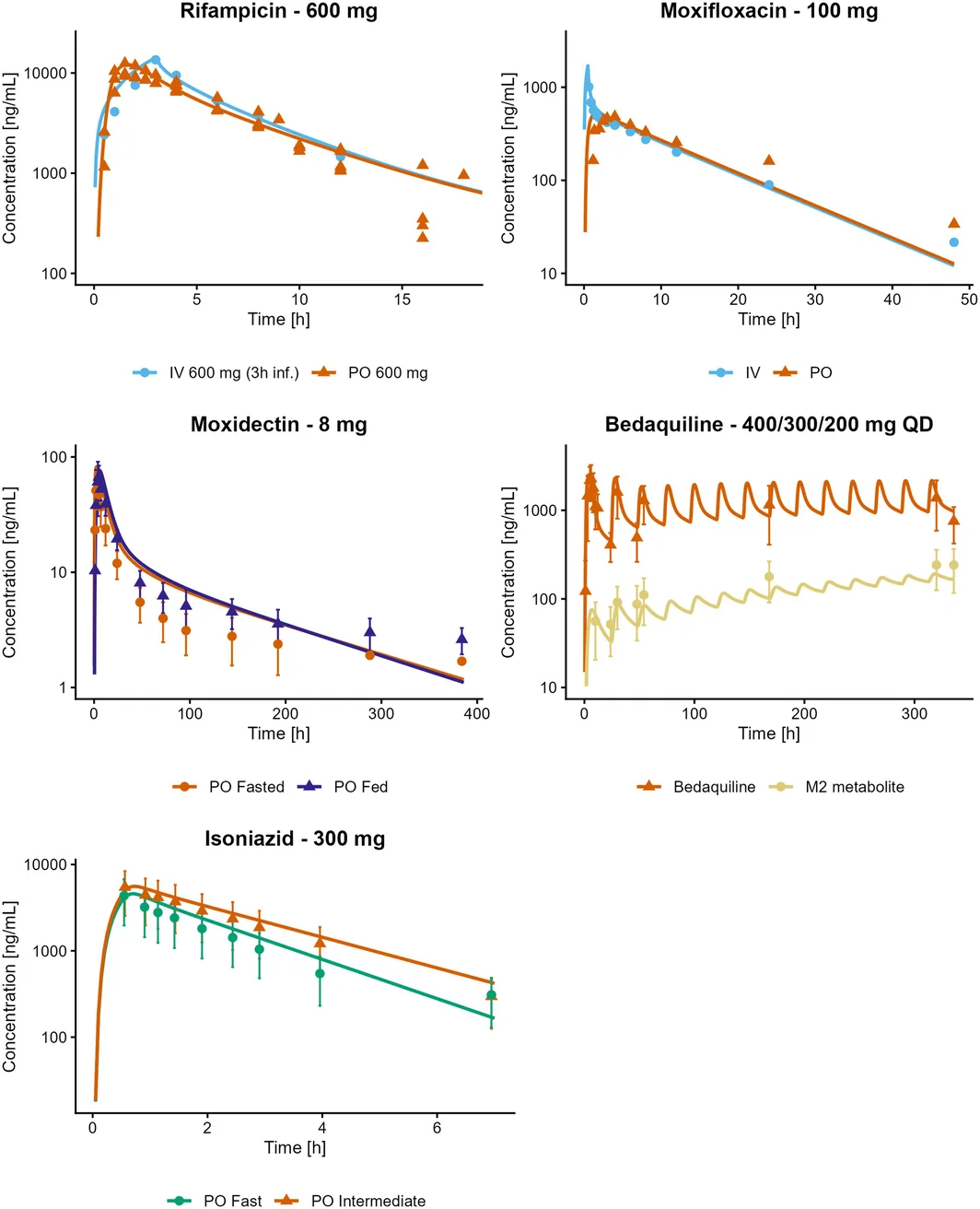
Predicted versus observed plasma pharmacokinetic profiles for rifampicin, moxifloxacin, moxidectin, bedaquiline, and isoniazid. Simulated concentration‐time profiles are shown for either an intravenous (IV) or an oral (PO) dose, in a fed or fasted state, for a fast or intermediate metabolizer for the given compounds and the N‐desmethyl bedaquiline (M2) metabolite. Observations were retrieved from the literature [13, 14, 15, 16, 17]. Observed data are represented as symbols and model predictions as lines.

Rifampicin was modeled across nine clinical studies covering intravenous and oral administration at doses of 300–600 mg, including scenarios reflecting its potent induction of CYP3A4 and CYP2B6 relevant to drug–drug interaction prediction. The model achieves a geometric mean fold error (GMFE) of 1.28 for both AUC and Cmax across 28 study arms, placing it within the accuracy range considered acceptable for regulatory submissions [18]. Isoniazid was modeled across 11 studies covering intravenous and oral dosing from 4.75 to 20 mg/kg and 300 to 900 mg, with explicit representation of fast and slow N‐acetyltransferase 2 acetylator phenotypes, achieving GMFE values of 1.23 for AUC and 1.30 for Cmax across 19 study arms. Bedaquiline was modeled across three oral dosing studies at 200 to 700 mg, reproducing both the parent compound and its pharmacologically active M2 metabolite, with GMFE of 1.13 for parent AUC and 1.14 for parent Cmax. Moxifloxacin was modeled across six studies covering intravenous and oral administration from 60 to 600 mg, with GMFE of 1.28 for AUC and 1.30 for Cmax across 15 study arms. Moxidectin was characterized across four oral studies at doses of 3–36 mg under fed and fasted conditions, achieving GMFE of 1.38 for AUC and 1.16 for Cmax across 15 study arms.

Across all five models, predictive performance falls within the range consistently reported for mechanistic PBPK models developed using commercial platforms and validated for regulatory purposes [5]. This confirms that open‐source tools can deliver regulatory‐grade accuracy, and that the quality of mechanistic PBPK modeling is not fundamentally dependent on access to proprietary software. The consistency of performance across compounds with diverse pharmacological profiles, ranging from the enzyme‐inducing rifampicin to the long half‐life tissue‐accumulating bedaquiline, provides confidence in the generalizability of the approach and its applicability to new TB candidates as they progress through development.4.3 Contribution to the integrated framework.

Mechanistic PBPK models serve as the connective tissue of the framework. They refine the generic approximations of HT‐PBPK for priority compounds, and their tissue concentration‐time outputs directly drive the cardiac electrophysiology and TB disease models, anchoring safety and efficacy simulations in physiologically realistic exposure conditions. The same infrastructure supports drug–drug interaction and special population analyses by adjusting physiological parameters to reflect organ impairment, comedication effects, or demographic differences, providing quantitative continuity across all modeling layers.

The drug–drug interaction modeling capability of the mechanistic PBPK layer is of particular relevance in the TB context. Many patients receiving TB treatment have concurrent conditions requiring other medications, including antiretroviral therapy for HIV co‐infection, oral contraceptives, diabetes medications, and cardiovascular drugs. The rifampicin model quantifies the relationships among rifampicin dose, CYP3A4 and CYP2B6 induction, and the resulting reductions in exposure of co‐administered substrates, enabling simulation of clinically relevant combination scenarios. As the model library expands to additional TB compounds, the same interaction framework can be applied to evaluate the net pharmacokinetic consequences of new three‐way or four‐way combinations, supporting pre‐clinical and clinical dose‐finding for regimens that include both established and investigational drugs.

## 5 Quantitative Assessment of Cardiac Safety

### 5.1 Scientific Rationale

Several anti‐TB agents, most prominently bedaquiline and moxifloxacin, are associated with QT interval prolongation and the potential for proarrhythmic effects. This cardiac liability represents a meaningful clinical consideration because TB treatment regimens frequently combine these drugs, ECG monitoring is not consistently available in the primary care and community settings where treatment is delivered in high‐burden countries, and patient populations often include individuals with underlying cardiac conditions, electrolyte imbalances, or concurrent medications that can compound electrophysiological risk. The standard approach of evaluating QT‐interval effects through dedicated thorough QT studies is resource‐intensive and cannot readily be applied prospectively to combination scenarios or to the range of patient subgroups relevant in practice. Mechanistic modeling of cardiac electrophysiology provides a cost‐effective, human‐relevant, and quantitatively informative alternative for characterizing and communicating these risks in a way that supports both clinical management and regulatory review [19].

The open‐source QSP‐PBPK cardiac safety framework developed as part of this platform predicts the electrophysiological effects of anti‐TB drugs by combining PBPK‐predicted systemic exposure with layered cardiac modeling components implemented in R. All resources are publicly available at https://github.com/esqLABS/Cardiac_Electrophysiology, accompanied by a practical how‐to guide and a scientific poster describing the methodology and results [20, 21]. The repository also provides step‐by‐step documentation for the full workflow, not only the electrophysiology example.

### 5.2 Framework Structure and Modeling Layers

The cardiac risk assessment workflow combines a shared pharmacokinetic layer with two independent, alternative approaches for translating exposure into electrophysiological risk, each suited to a different stage of development and level of data availability. The first layer consists of the mechanistic PBPK models described in the preceding section, which predict free plasma drug concentrations under defined dosing regimens, including standard therapy doses and scenarios involving drug–drug interactions, metabolic impairment, or population variability. Free concentrations, rather than total plasma concentrations, are used as the pharmacokinetically active input because unbound drug is responsible for ion‐channel interaction and because protein binding varies meaningfully across compounds and patient characteristics. The second layer is a data‐driven QTc model adapted from the framework of Gotta and colleagues [22], which translates free drug or metabolite concentrations into hERG‐mediated QTc prolongation. The model uses in vitro hERG potassium channel inhibition data to describe compound‐specific channel blockade, and applies a pharmacokinetic‐pharmacodynamic transduction relationship to estimate the resulting QTc response. This relationship separates human system‐specific properties, such as maximal hERG‐mediated QTc prolongation and response steepness, from a compound‐specific parameter describing how efficiently hERG block is converted into QTc prolongation. Estimation of the compound‐specific parameter requires in vivo exposure–QTc information, which may be obtained from preclinical cardiovascular safety studies combined with species‐to‐human scaling, as in the original Gotta framework, or directly from clinical concentration‐QTc data. In the present application, suitable preclinical QTc data were unavailable, so the compound‐specific transduction parameters were calibrated directly against publicly available clinical exposure‐QTc data [23, 24]. The third layer (alternative to the second layer) is a mechanistic cardiac electrophysiology model translated from MATLAB to R on the basis of the action potential model of Llopis‐Lorente and colleagues [25]. This model is derived from the O'Hara‐Rudy (ORd) model; it predicts cardiomyocyte action potentials and calculates several biomarkers such as action potential duration at 90% repolarization (APD90) in virtual male and female subject populations, which differs by myocardial ion channel densities and the effect of hormones on ion currents [25].

The PBPK layer enables simulation of exposure under realistic clinical conditions, including variable free concentrations resulting from protein binding variability, metabolic phenotype differences, and drug–drug interactions, rather than relying on average or assumed concentration inputs. The QTc layer links in vitro hERG potency and free drug or metabolite exposure to predicted clinical QTc prolongation. Compound‐specific prediction requires in vivo exposure‐QTc data to identify the transduction from hERG block to QTc effect. Such data may come from preclinical cardiovascular safety studies or directly from clinical PK‐matched ECG data. A dedicated thorough‐QT study is not mandatory, provided that a SAD, MAD, or population PK‐QTc dataset adequately covers the relevant exposure range. In the present implementation, suitable preclinical data were unavailable, so moxifloxacin and bedaquiline/M2 were calibrated directly to clinical thorough‐QT and population PK‐QTc data, respectively. Once calibrated, the model can characterize QT liability and simulate alternative dose, exposure and metabolite scenarios. The mechanistic electrophysiology layer addresses this by simulating the cardiomyocyte action potential under drug effect based on in vitro patch‐clamp assay data, capturing the non‐linear concentration‐response relationships and potential interaction effects across multiple ion channels that simpler models cannot resolve. Because this layer requires only in vitro channel‐inhibition data and a free plasma concentration estimate, which can be obtained from any PBPK approach, including QSAR‐predicted exposure from the HT‐PBPK pipeline (Section 3), it is, in principle, applicable even before (pre)clinical pharmacokinetic or QT data become available, unlike the data‐driven QTc layer described above. In the case studies presented here, free plasma concentrations were taken from the validated mechanistic PBPK models; earlier application using HT‐PBPK‐predicted exposure is a planned extension of the framework (Section 5.4). Ideally, IC50 values should be informed by in vitro assays, as per the recommendation of the FDA Comprehensive In vitro Proarrhythmia Assay (CiPA) initiative [26]. The fractional current inhibition should be plotted against the tested drug concentration, with increasing concentrations and a Hill function should be fitted to the data to derive IC50 and a hill coefficient (non‐linear least square algorithm).

### 5.3 Calibration, Validation and Illustrative Results

The PBPK models driving the cardiac framework reproduced observed plasma pharmacokinetics for the five anti‐TB drugs, with an overall geometric mean fold error of approximately 1.1, confirming the reliability of the free‐concentration inputs to the cardiac modules. The data‐driven QTc model captured the observed concentration‐dependent QTc signal for moxifloxacin, with predicted QTc prolongation increasing across the clinical concentration range and remaining in the range of the reported thorough QT data [23] (Figure 3A). For bedaquiline, the model captured the exposure‐dependent relationship between M2 concentrations and QTc, with a rapid increase at lower M2 concentrations followed by an apparent plateau at higher concentrations [24] (Figure 3B). These results demonstrate that the QTc component of the framework can represent established compound‐specific exposure‐QTc relationships and therefore support simulations of alternative exposure, dose, and interaction scenarios. It should be noted that these results demonstrate calibration performance and not independent predictive validation.

**FIGURE 3:**
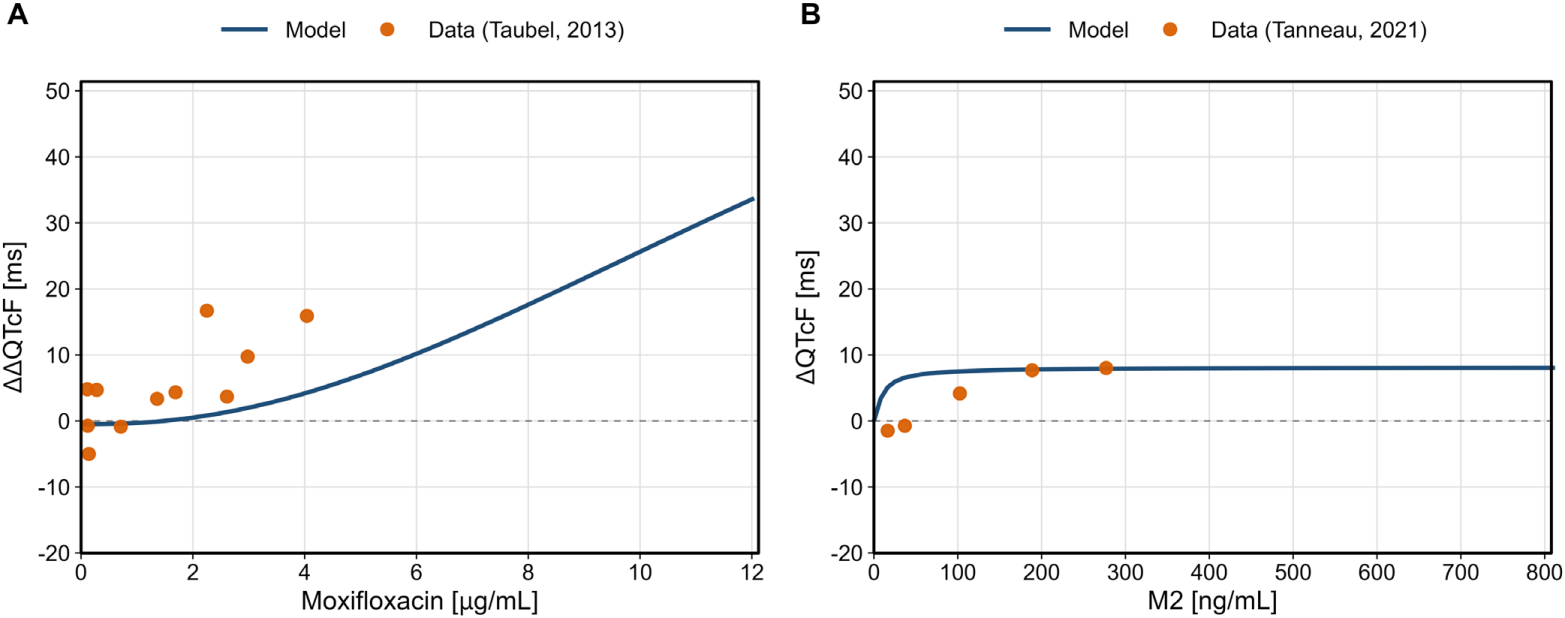
Comparison of the data‐driven QTc model predictions with published concentration–QTc data for moxifloxacin (panel A) and bedaquiline/M2 (panel B) (Taubel [23]; Tanneau [24]). Fridericia correction was applied using a resting heart rate of 75 bpm. Change from baseline QTcF for placebo was assumed to be 0.5 ms.

The R implementation of the electrophysiology model was validated against the CiPA benchmark drug set, successfully reproducing the action potential changes described in the original publication [25] when using the same input conditions. For example, Dofetilide‐induced APD90 prolongation was correctly reproduced in males and females using hERG IC50 of 1.47 nM as an inhibitory potential and no inhibition (high IC50) for the other channels (Figure 4A–E). The model was also able to reproduce the expected repolarization abnormalities in females treated with quinidine using free plasma concentration of 1258.8 nM, consistent with the known proarrhythmic risk of this compound and the results of the original model (Figure 4F). Forward predictions were then done for the TB drugs bedaquiline and moxifloxacin using as input the free plasma concentration predicted by the aforementioned PBPK models. For bedaquiline, which has a reported hERG IC50 of 1.6 μM, no electrophysiological effect was predicted at free plasma concentrations achieved under standard therapeutic dosing of 400 mg followed by 300 mg and 200 mg weekly. However, simulations of a hypothetical scenario in which unbound plasma concentrations were raised to 500 nM, as could occur under conditions of hepatic impairment or co‐administration of CYP3A4 or CYP2C8 inhibitors, predicted an approximately 14% increase in APD90 in male virtual subjects (Figure 5A,B). For moxifloxacin, whole‐cell patch clamp data [27] were used to derive IC50 values of 94.4, 288.3, 1502.9, and 52.9 μM for hERG (IKr), Late sodium (iNaL), L‐type calcium (iCaL), and Slow delayed rectifier potassium current (iKs), respectively. Of note, the inhibitory potential was slightly overestimated compared to data for iKs, and no data were available for the fast sodium current (iNa), no inhibition was assumed (high iNa IC50). The PBPK model predicted an input unbound plasma concentration of 8.4 μM at the dosing of a published clinical trial (60‐min iv infusion at 5.63 mg/h/kg followed by 30‐min at 0.26 mg/h/kg [27]), the M2 metabolite concentration was estimated to be 5.8 μM, assuming 40% of parent concentration and 5% binding [27]. The parent and metabolite were assumed to bind at the same binding site and have a similar potency (IC₅₀), as observed for hERG channels [27]. The cardiac electrophysiology model predicted that moxifloxacin significantly increased APD90 by 35 ms under the tested clinical dose compared to control (Figure 5C,D), in line with the reported ΔΔQTc of 29.9 ms [27].

**FIGURE 4:**
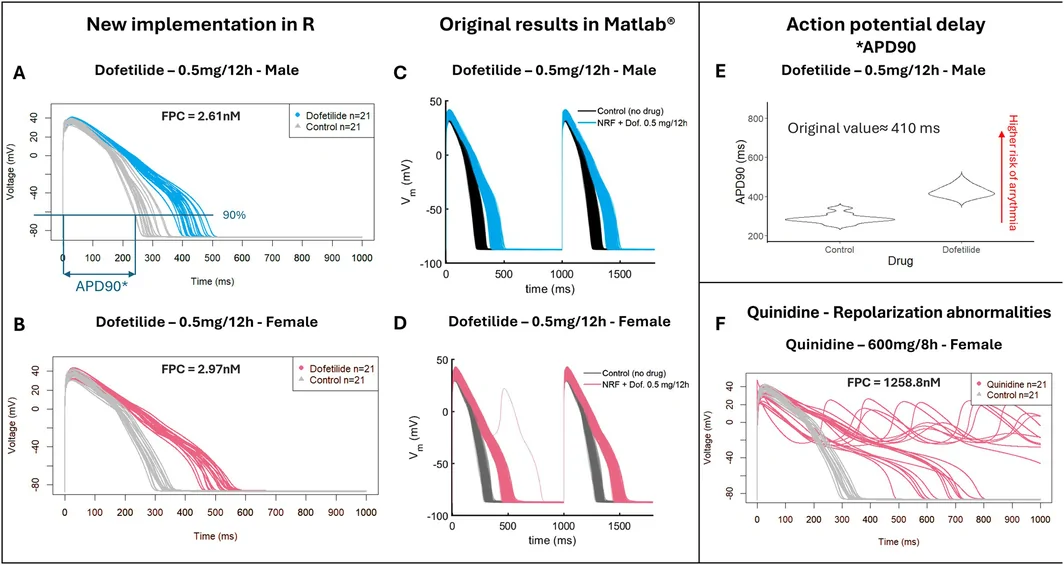
Validation of the R‐based cardiac electrophysiology model implementation against CiPA benchmark compounds. Action potential predictions from the R implementation are compared with reference results from the original published model [25]. Dofetilide was modeled with IC50, hERG = 1.47 nM, reproducing characteristic action potential duration (APD90) prolongation consistent with its known proarrhythmic risk. Quinidine was modeled with IC50 of 340 nM and 9460 nM for hERG and iNaL, respectively, reproducing the repolarization abnormalities from the original paper in females. FPC, free plasma concentration.

**FIGURE 5:**
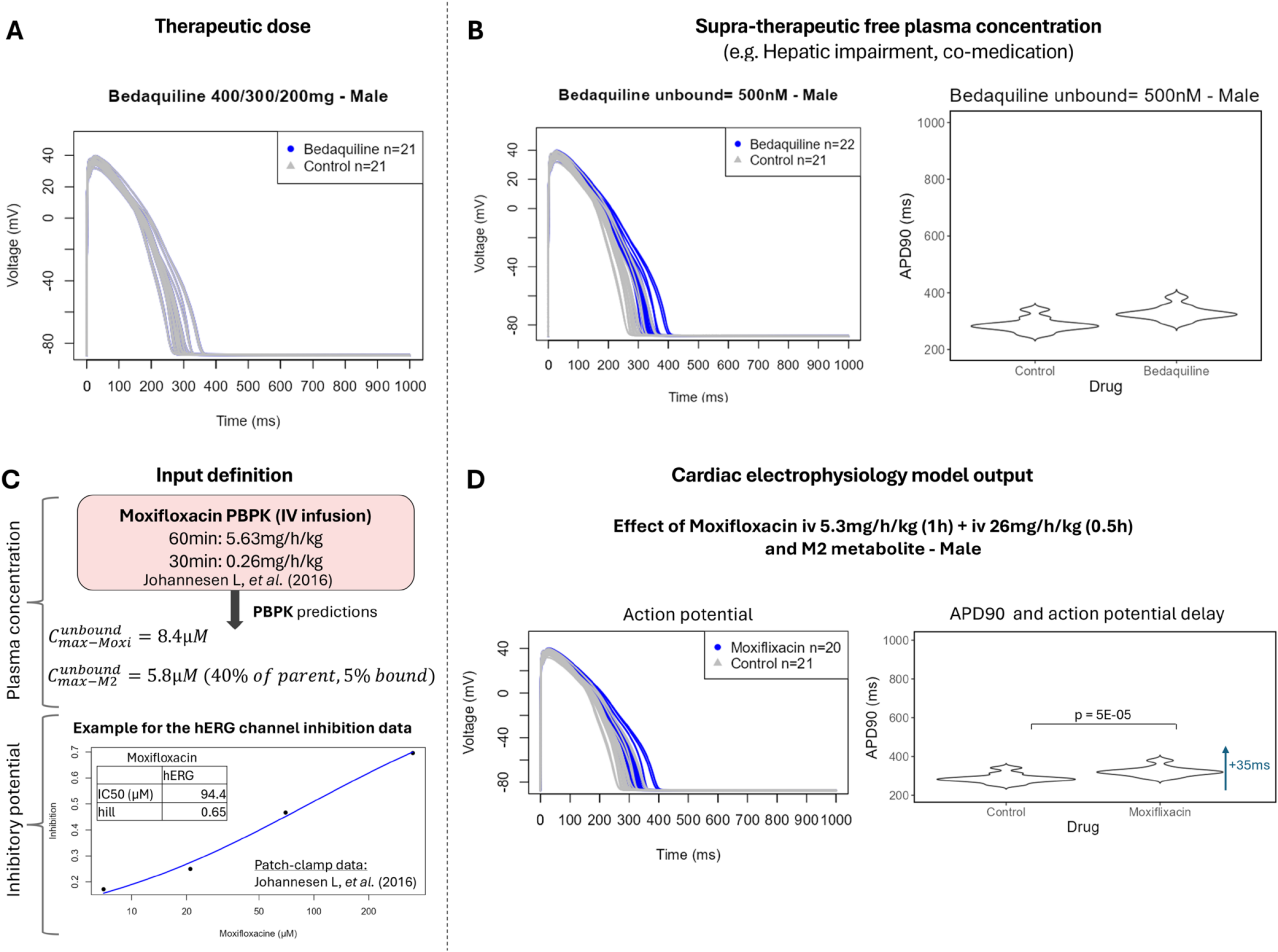
Predicted effect of bedaquiline and moxifloxacin on the cardiomyocyte action potential. (A) Bedaquiline effect under standard therapeutic dose and (B) under elevated free plasma concentration (500 nM). *N* = 22 and 21 for treatment and control, respectively. (C) Input free plasma concentration of moxifloxacin (Moxi) was obtained from the PBPK simulations under a published clinical trial dosing regimen; input IC50 and hill coefficient values were obtained from fitting channel inhibition patch‐clamp assay data for hERG (IKr), late sodium (iNaL), L‐type calcium (iCaL) and slow delayed rectifier potassium current (iKs). (D) Effect of Moxifloxacine and M2 metabolite on the cardiomyocyte action potential in males (*n* = 20 and 21 for treatment and control, respectively). Student *t*‐test was used to assess the significance of the effect.

### 5.4 Interpretation and Position Within the Framework

Cardiac safety assessment for anti‐TB drugs is achievable within a fully open‐source environment, and these results demonstrate it. Deriving a quantitative relationship between the action potential delay and QTc prolongation would be beneficial for further validation and clinical assessment of the output. Operating in parallel with the TB disease model (c.f. Section 6), this framework supports concurrent efficacy‐safety evaluation under realistic dosing conditions. Its value is greatest for combination regimens, where the assumption of independent single‐drug cardiac risk is insufficient and where simulated exposure variability across patient subgroups matters most. The two downstream layers are intended as independent, alternative paths rather than a fixed sequence. The data‐driven QTc layer can be applied once compound‐specific in vivo exposure‐QTc data are available, and support quantitative evaluation of dose, exposure, and metabolite scenarios. In contrast, the mechanistic electrophysiology layer can be applied earlier in development, using in vitro ion‐channel data together with a predicted or measured free plasma concentration, to flag candidates that warrant closer safety evaluation before in vivo QTc data are available. Planned extensions include multi‐channel ion data beyond hERG, earlier integration with the HT‐PBPK pipeline, and direct coupling with the disease model to test whether efficacy‐driven dose adjustments shift cardiac risk profiles.

The level of clinical data needed to apply the data‐driven QTc layer follows directly from its structure. Because the human system parameters and the in vitro hERG potency estimate are fixed, only a single compound‐specific transduction parameter must be calibrated, so one sufficiently informative concentration‐QTc dataset can identify it. A single SAD or MAD study is adequate when it provides ECGs matched closely in time to measured concentrations or reliable individual PK predictions, baseline‐adjusted QTc measurements with, preferably, a placebo comparison, and several exposure levels spanning the therapeutic and clinically relevant high‐exposure ranges. SAD data may suffice for a directly acting parent compound without important accumulation or delayed effects [28, 29], whereas MAD data are preferable when the relevant exposure occurs only at steady state or when an active metabolite accumulates or forms with delay, as for bedaquiline and its M2 metabolite [24]. Multiple studies are not intrinsically required, but become valuable to extend exposure coverage, separate parent and metabolite contributions, or provide independent validation; this is consistent with ICH E14/S7B guidance that concentration‐QTc data need not arise from a dedicated QT study nor necessarily from a single study [30]. Suitable preclinical exposure‐QTc data can also be used with species‐to‐human scaling, as in the original framework [22].

The clinical implications of these findings extend beyond individual compound assessment. TB treatment guidelines increasingly recognize the need to monitor QTc in patients receiving bedaquiline and moxifloxacin together, and in patients with pre‐existing cardiac risk factors [31]. The QSP‐PBPK framework can support the design of monitoring algorithms and dose adjustment thresholds by predicting how QTc‐interval prolongation or action potential delay change as a function of exposure, co‐medication, and patient characteristics. It could also provide quantitative input for regulatory benefit–risk assessments by translating predicted changes in APD90 into probabilistic estimates of arrhythmia risk, using relationships established from in vitro and clinical data. As the CiPA initiative related frameworks continue to develop and validate multi‐channel electrophysiology models, the open‐source architecture of this framework allows the cardiac module to be updated with improved channel parameterizations without rebuilding the PBPK or cardiac effect components.

## 6 Mechanistic Tuberculosis Disease Model

### 6.1 Design Rationale and Overall Structure

The mechanistic TB disease model forms the systems‐level pharmacology component of the integrated framework. Its purpose is to translate drug exposure predictions from the PBPK models into biologically and clinically meaningful outcomes, including bacterial killing dynamics, immune‐mediated control of infection, granuloma sterilization, and long‐term treatment trajectories. The model was developed as a comprehensive and transparent reconstruction of the published Fors et al. [32] model in MoBi, reorganized into a modular structure that supports flexible extension, clear parameterization, and direct coupling with upstream PBPK models and parallel safety modules. The full model implementation, documentation, and technical instructions for running simulations are openly available at https://github.com/esqLABS/Tuberculosis‐model, providing the information needed for independent reproduction, verification, and adaptation by other research groups. An overview of the model architecture is given in Figure 6.

**FIGURE 6:**
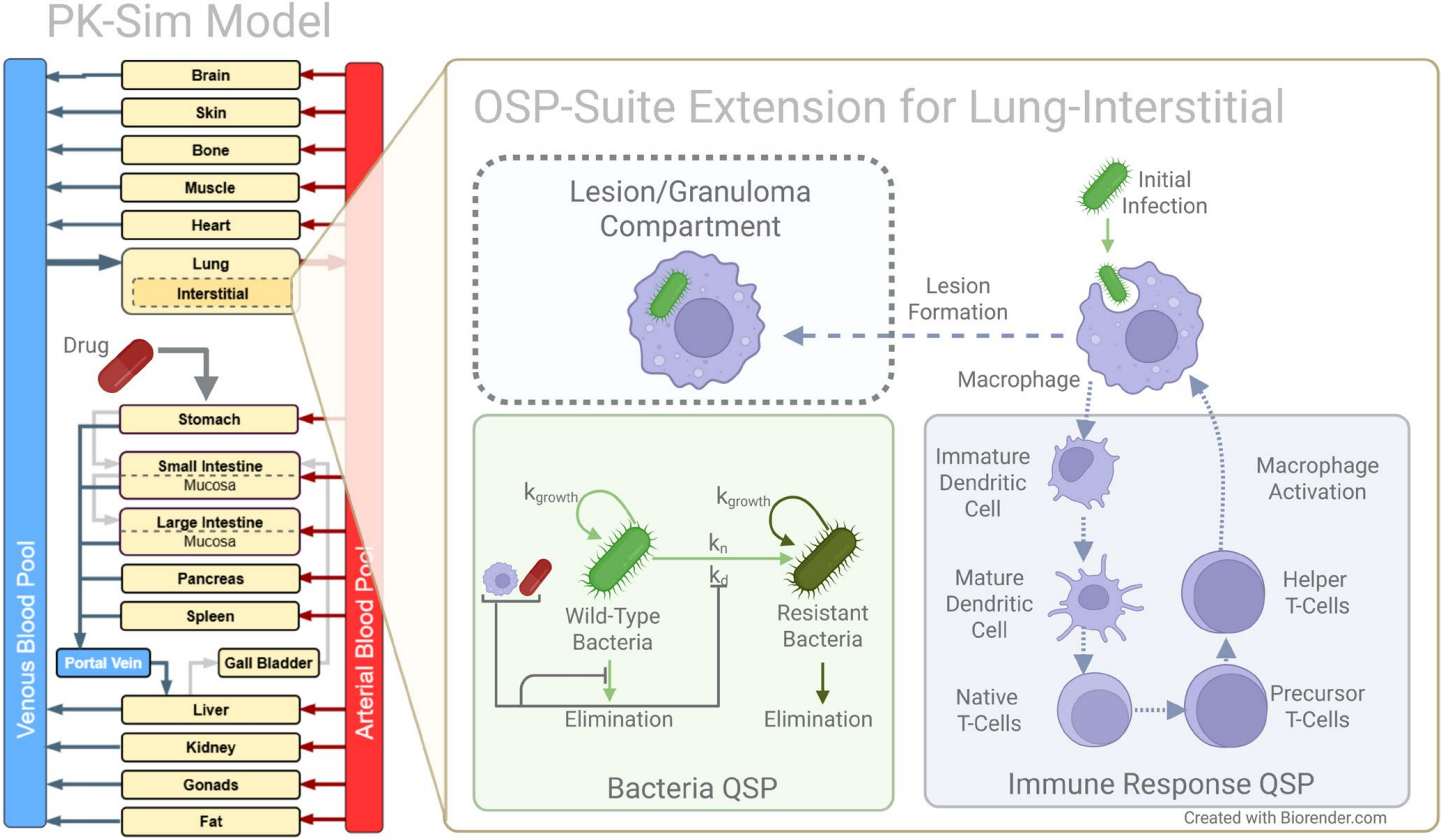
Architecture of the mechanistic TB disease model integrated with whole‐body PBPK. Drug concentrations predicted by PK‐Sim in lung interstitial fluid drive pharmacodynamic effects within the disease model. Partitioning into granulomatous lesions, macrophages, and intracellular compartments follows OSP tissue distribution models and the Fors et al. [32] framework. The disease module captures lesion dynamics, immune activation, and bacterial behavior across extracellular and intracellular niches for both wild‐type and drug‐resistant subpopulations. Adapted from Fors et al. [32]. This figure was created in Biorender. Albrecht, M.

The biological representation centers on pulmonary TB infection and describes the key spatial and cellular compartments where drug activity takes place. The model contains two spatial domains: the lung interstitium and the granulomatous lesion. Within each domain, both extracellular and intracellular bacterial populations are represented alongside migrating immune cells and drug effects that vary across compartments, depending on the physicochemical properties of each compound and the physiological characteristics of each microenvironment. This compartmentalization reflects the biological reality of TB infection, in which subpopulations of *M. tuberculosis* occupy distinct niches with different oxygen tensions, pH values, metabolic activity levels, and drug accessibility. These differences are directly relevant to treatment outcomes because drugs effective against rapidly replicating extracellular bacteria may have limited activity against dormant or slowly replicating intracellular populations, and the sterilizing activity necessary to prevent relapse depends on reaching and killing bacteria in all relevant compartments.

### 6.2 Biological Components and Mechanisms

Within the modeled compartments, bacterial growth, phenotypic transitions between metabolic states, and interactions with macrophages are described by coupled differential equations. The model captures the behavior of bacteria residing inside macrophages and within the hypoxic granuloma core, both of which are associated with phenotypic tolerance to drug treatment and with persistence following apparent clinical cure. Macrophage activation, recruitment, and immune‐mediated killing of intracellular bacteria are represented, together with a feedback relationship between bacterial burden and the intensity of the immune response. These dynamics allow the model to simulate the natural history of TB infection, from an initial inoculum through early uncontrolled growth to immune‐mediated plateau and transition to chronic infection.

Drug effects are implemented as submodular extensions added to the core biological structure. Each compound receives its own pharmacodynamic effect module, specifying the mechanism of action, the targeted compartment or bacterial population, and the concentration‐effect relationship. This architecture allows rapid testing of new drug candidates or modified dosing scenarios without altering the underlying disease dynamics. Because the submodules are independent additions, they can be modified or replaced to reflect updated pharmacodynamic parameter estimates as experimental data accumulate, and new compounds can be introduced without rebuilding the biological core. Wild‐type and drug‐resistant bacterial subpopulations are tracked simultaneously, with resistance defined through strain‐specific growth parameters and mutation rates, enabling simulation of resistance emergence and amplification under drug selection pressure.

### 6.3 Integration With PBPK Exposure Predictions

Direct integrability with whole‐body PBPK simulations generated in PK‐Sim is one of the model's most practically useful properties. Concentration‐time profiles predicted by mechanistic PBPK models are used as inputs to the disease model to drive drug effects in the lung interstitium, granulomatous lesions, and intracellular macrophage compartments. Drug partitioning into the lung interstitium and into lesion and intracellular spaces follows tissue‐specific models based on OSP physiological templates and the Fors et al. [32] framework, ensuring that the simulated drug exposure in each biological niche reflects the compound‐specific properties, including lipophilicity, membrane permeability, and intracellular binding, that govern local penetration. An overview of the effect model simulations is given in Figure 7. This direct PBPK‐to‐disease model coupling creates a fully connected simulation environment from administered dose through systemic and tissue pharmacokinetics to bacterial response, supporting physiologically consistent simulation of regimen scenarios that can incorporate the variability and uncertainty in each modeling step.

**FIGURE 7:**
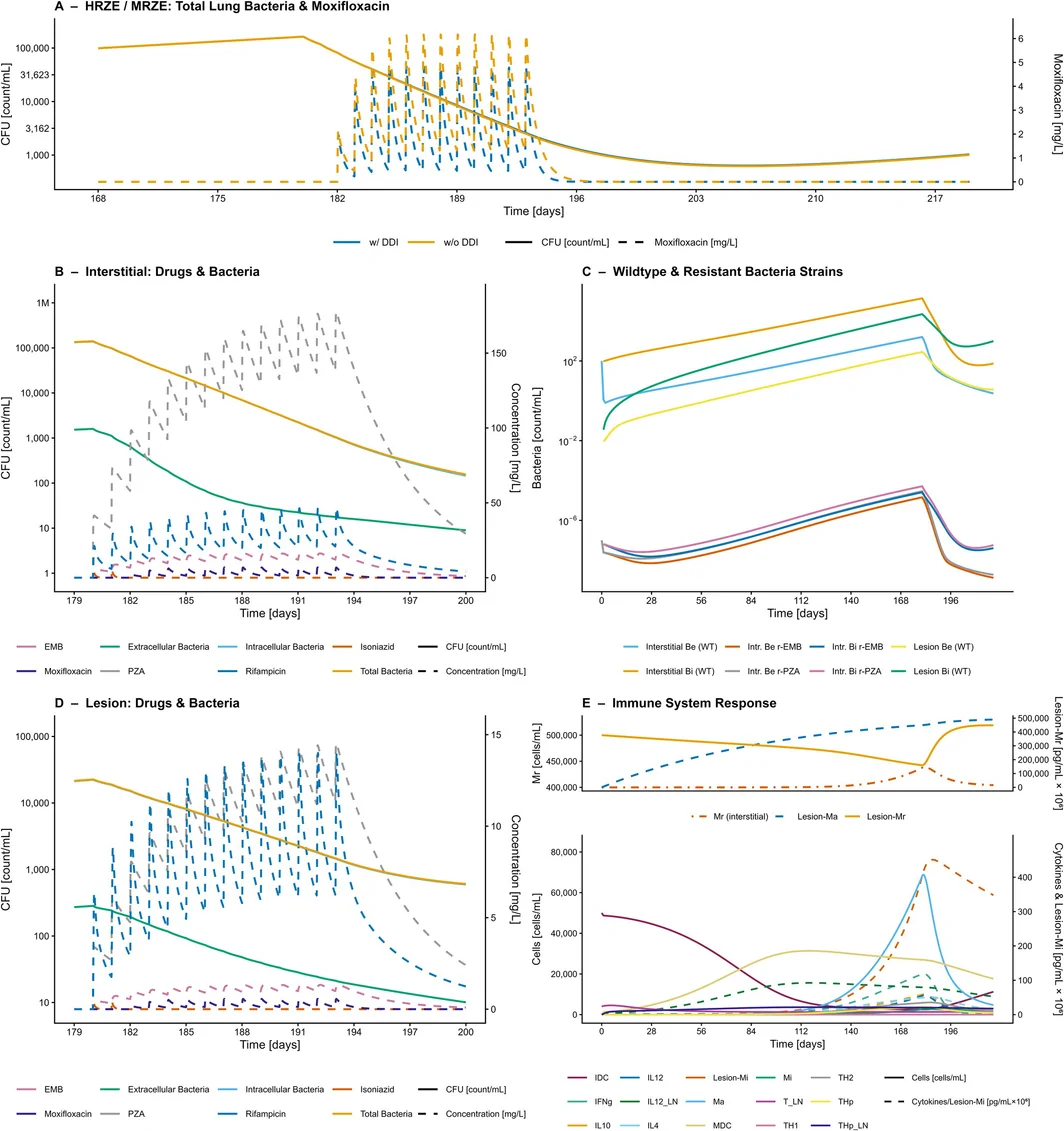
Integrated simulation of multi‐drug pharmacokinetics, immune response, and bacterial dynamics across lung and lesion compartments. (A) Effect of the rifampicin‐moxifloxacin drug–drug interaction (DDI) on simulated lung bacterial burden. Bacterial dynamics under HRZE‐MRZE regimens are compared with and without rifampicin‐mediated reduction of moxifloxacin exposure. (B) Predicted drug concentrations in lung interstitial fluid following dosing. (C) Trajectories of wild‐type and resistant bacterial subpopulations. (D) Predicted concentrations in lesion compartments. (E) Immune system dynamics. Additive drug effects progressively reduce bacterial burden across all compartments.

The coupled structure also supports translation across populations and clinical scenarios. Because the exposure inputs come from mechanistic PBPK models that can be parameterized for different patient groups, the disease model outcomes automatically reflect the pharmacokinetic differences associated with metabolic phenotype, organ impairment, drug–drug interactions, or demographic characteristics without requiring separate reparameterization of the disease dynamics. This allows the framework to address population‐level questions, such as the distribution of bacterial outcomes expected across a clinical trial population or the differential response of fast and slow acetylators to isoniazid‐containing regimens, in a structurally consistent way.

### 6.4 Verification and Comparison With Published Behavior

The MoBi reimplementation was verified against the original C++ model of Fors et al. [32] and across the approximately 50 scenarios reported in that publication and its supplementary material, covering untreated infection, immune‐mediated bacterial control, monotherapy with each of the included drugs, combination regimens, early bactericidal activity windows, and long‐term sterilization trajectories. Key dynamic patterns, including uncontrolled bacterial growth in untreated infection, gradual immune‐mediated transition to a bacterial plateau, and the characteristic treatment response under drug pressure, were reproduced with close qualitative and quantitative agreement between the two implementations. The patterns of intracellular and extracellular bacterial burden over time were consistent across both models, with intracellular populations rising more rapidly to an earlier plateau while extracellular populations expanded later and ultimately dominated total bacterial load.

Pharmacodynamic patterns characteristic of the included drugs, consistent with those reported in the same Fors et al. [32] reference model described above, were also reproduced across treatment simulations. Rifampicin's dominant sterilizing effect in combination regimens is clearly reflected in model output, consistent with its established clinical role as the primary bactericidal and sterilizing agent in first‐line therapy. Moxifloxacin and isoniazid reproduce the expected differences in early bactericidal activity, and bedaquiline shows a delayed but sustained reduction in bacterial burden, consistent with its mechanism of targeting the mycobacterial ATP synthase and its known clinical pharmacodynamic profile. Although early bactericidal activity values tend to be conservatively estimated relative to published clinical data in some cases, the relative ranking and qualitative behavior of drugs across simulated scenarios is consistent with clinical pharmacological interpretation, indicating that the model structure captures the relevant dynamics of drug action reliably. Further refinement of bactericidal and bacteriostatic parameters is expected to improve quantitative agreement as new in vitro and clinical data are incorporated.

### 6.5 Scenario Exploration and Applications

The model supports a broad range of analyses relevant to TB drug development and clinical research. It can simulate dose–response relationships across a defined dose range, evaluate the consequences of regimen shortening on sterilization trajectories, model the impact of imperfect adherence on bacterial dynamics and resistance risk, and assess the effects of pharmacokinetic variability associated with metabolic phenotypes or organ function changes. Because both intracellular and extracellular bacterial populations in lung and lesion compartments are tracked simultaneously, the model is well‐suited to examine questions about drug penetration into pharmacologically challenging environments, relative contributions of different drug classes to sterilization and resistance suppression, and the biological consequences of exposure‐dependent effects on the slow‐replicating persistent population.

The model has been applied to explore standard HRZE and RZE induction regimens, moxifloxacin‐containing combination scenarios relevant to drug‐sensitive and drug‐resistant TB, bedaquiline in monotherapy and combination settings, and the hypothetical application of moxidectin as a candidate compound. A case simulation examining the rifampicin‐moxifloxacin drug–drug interaction illustrates how the framework can evaluate interaction‐mediated changes in drug exposure against bacterial outcome endpoints. Under standard treatment conditions, the difference in bacterial burden between the interaction and no‐interaction scenarios is modest, reflecting rifampicin's dominant sterilizing activity. The effect of the interaction becomes more pronounced in simulation scenarios where rifampicin‐resistant bacterial subpopulations are present and moxifloxacin plays a larger independent role in bacterial control, a scenario directly relevant to the treatment of fluoroquinolone‐susceptible drug‐resistant TB.

An important application of the disease model is the prospective evaluation of novel drug candidates before clinical data are available. When a compound exhibits characterized PBPK behavior but lacks clinical pharmacodynamic data in TB, the model can generate preliminary predictions of antibacterial activity in lung and lesion compartments using mechanistic PBPK output as exposure input and available in vitro MIC and kill‐rate data as pharmacodynamic parameters. Although such predictions carry substantial uncertainty, they provide a structured basis for prioritizing candidates, selecting dose levels for early clinical evaluation, and designing pharmacokinetic‐pharmacodynamic sampling strategies in early phase trials. The modular architecture ensures that, as clinical data become available, they can be incorporated to refine the pharmacodynamic submodule, improving predictive accuracy without restructuring the disease component. This capacity for iterative refinement is a key feature of the framework and reflects the tutorial principle that modeling should evolve alongside the evidence rather than being a one‐time exercise.

### 6.6 Limitations and Future Development

The model performs well qualitatively across a broad range of scenarios, but several aspects require further refinement before quantitative predictions can be used with high confidence for clinical decision support. Drug effect submodules need optimization of bactericidal and bacteriostatic parameters to fully reproduce early bactericidal activity data for all compounds. Persistence mechanisms governing the slow‐replicating bacterial population require more detailed parameterization based on experimental measurements of phenotypic tolerance. Lesion and macrophage transport parameters should be updated as emerging imaging, histological, and pharmacokinetic data provide more precise characterization of the relevant tissue microenvironments. The immune module captures the broad dynamics of macrophage‐mediated control but would benefit from a more detailed representation of phenotypic differences in immune response, particularly in scenarios involving HIV co‐infection or immunosuppression from other causes.

Model developments include integration of resistance evolution models to characterize the probability and time course of resistance emergence under different drug selection pressures, addition of vaccine‐related immune phenotypes to support the analysis of TB vaccine candidates in combination with drug therapy, and expansion to population PBPK‐coupled simulations that can characterize the distribution of individual treatment outcomes across a patient population defined by relevant demographic and genetic characteristics. These extensions will progressively broaden the practical scope of the framework and increase its utility across the full spectrum of decisions facing TB drug developers, from early compound selection and dose‐ranging to late‐stage regimen optimization and regulatory submission.

## 7 Discussion

The framework presented in this tutorial demonstrates a fully integrated, open‐source modeling platform for TB drug development that can find application across different phases of drug development. Each of the four components, taken individually, addresses a well‐defined pharmacological need in TB pharmacology. Taken together, they create a connected system that can accompany a compound from the earliest stages of characterization through the quantitative questions in translational and clinical development. The critical enabling factor is not the complexity of any individual model but the design principle of interoperability: each component is built to receive inputs from and pass outputs to the others within a shared, open infrastructure.

Compared with existing approaches that rely on proprietary platforms, the open‐source framework offers three practical advantages relevant to TB drug development. First, full transparency: every equation, parameter, and assumption is accessible for inspection, and the entire analytical path from input data to model output can be traced and audited. This is increasingly important as regulatory agencies and scientific journals raise expectations for reproducibility and for clear documentation of modeling decisions [18]. Second, adaptability: because the models are distributed as editable code rather than locked software objects, they can be extended to new compounds, updated to incorporate new data, and modified to address new scientific questions without waiting for a software vendor update. Third, accessibility: the framework can be used without institutional licenses or commercial subscriptions, lowering barriers to participation in model‐informed TB research in academic, governmental, and low‐resource settings. This parity applies across all four components of the framework, not only HT‐PBPK: it reduces the systematic bias that would otherwise arise from high‐burden settings being unable to contribute model‐informed evidence to global TB drug development priorities.

The performance benchmarks achieved by the mechanistic PBPK models across five structurally diverse TB drugs are consistent with those reported for models developed using commercial platforms. GMFE values in the range of 1.1–1.4 compare favorably with published evaluations of PBPK models built in other commercial platforms for the same or similar compounds [5]. This level of agreement provides practical support for the use of open‐source tools in regulatory and translational settings. It suggests that, when models are appropriately qualified and documented, predictive accuracy comparable to that of commercially developed PBPK models can be achieved within a fully open framework. As the evidence base for open‐source PBPK modeling continues to grow, this may support broader acceptance of such approaches, particularly in contexts where transparency, reproducibility, and open accessibility are valued.

The cardiac safety component of the framework addresses a gap in existing TB modeling practice. Most published PBPK models for TB drugs do not include a systematic cardiac electrophysiology module, and cardiac risk in TB combination therapy is typically assessed by reviewing individual compound safety data rather than by prospective quantitative integration. The QSP‐PBPK approach presented here enables simulation of proarrhythmic risk under conditions difficult to study experimentally, including long‐term dosing with multiple QT‐affecting compounds, exposure changes due to drug–drug interactions, and the additional risk associated with specific patient characteristics. These are precisely the scenarios of greatest clinical relevance in drug‐resistant TB treatment, where bedaquiline is combined with other QT‐prolonging agents such as clofazimine and fluoroquinolones in patients who may already have compromised cardiac reserve. Extending the cardiac module to cover these multi‐drug combination scenarios is a priority for future development. This clinical combination‐therapy use case is addressed by the data‐driven QTc and the mechanistic electrophysiology layers. In addition, the mechanistic electrophysiology layer offers a route to early, discovery‐stage cardiac de‐risking for novel candidates, using in vitro channel data and predicted exposure before clinical pharmacokinetic data exist, complementing rather than duplicating the data‐driven layer's clinical focus.

The TB disease model contributes a dimension that pharmacokinetic modeling alone cannot provide: a mechanistic connection between drug exposure and the clinical outcomes that determine treatment success or failure. The ability to simulate bacterial killing, lesion sterilization, and resistance dynamics under physiologically realistic exposure conditions bridges the gap between a drug's pharmacological properties and its predicted clinical utility. This connection is valuable for communicating the translational relevance of modeling predictions to clinical and regulatory audiences that are more familiar with bacteriological endpoints than pharmacokinetic metrics. It also provides a quantitative framework for discussing regimen‐level questions, such as whether a shorter regimen is likely to achieve equivalent sterilization if exposure is optimized, or whether a resistance‐associated mutation that reduces susceptibility by a defined factor is likely to compromise treatment success under standard dosing.

Several limitations of the current framework should be acknowledged. The disease model parameters for some pharmacodynamic endpoints, particularly early bactericidal activity, are not yet fully optimized to reproduce quantitative clinical data across all drugs, and predictions of absolute bacterial killing rates should be interpreted with appropriate caution. The HT‐PBPK component performs best for compounds with moderate lipophilicity and good aqueous solubility, and its accuracy decreases for highly lipophilic or poorly soluble molecules where generic absorption and distribution models are less reliable. The cardiac electrophysiology module currently focuses on hERG‐mediated QT prolongation and does not explicitly model other ion channels that contribute to arrhythmia risk in specific patient subgroups or under specific interaction scenarios. These limitations do not undermine the utility of the framework for the intended purposes described in this tutorial, but they define the scope within which each component should be applied and highlight the areas where experimental data most clearly limit predictive confidence.

## 8 Conclusions

This tutorial has presented an integrated, open‐source, modular framework for anti‐TB drug modeling that spans the full development continuum from early compound screening through mechanistic pharmacokinetic modeling, pharmacodynamic assessment, and quantitative cardiac safety evaluation.

The framework is built entirely on the Open Systems Pharmacology Suite and the R computing environment, and all components are accessible through public repositories with documentation sufficient for independent reproduction, verification, and extension.

The high‐throughput PBPK component provides a scalable early‐stage screening tool capable of generating preliminary exposure estimates for large compound libraries using predicted or sparse experimental inputs. Application to 12 anti‐TB compounds demonstrated performance consistent with published benchmarks for generic PBPK, confirming its practical value for early compound triage and prioritization. The mechanistic PBPK models for rifampicin, isoniazid, moxifloxacin, bedaquiline, and moxidectin reproduce observed clinical pharmacokinetics within regulatory‐grade accuracy across diverse dose ranges, routes of administration, and population characteristics, establishing that open‐source platforms can deliver the quantitative reliability required for translational and regulatory applications.

The cardiac safety framework connects PBPK‐predicted drug concentrations to cardiac electrophysiology through two independent, alternative approaches: a data‐driven QTc model that combines in vitro hERG potency with compound‐specific in vivo exposure‐QTc information, and a mechanistic action potential model that runs on in vitro channel data and can support earlier, discovery‐stage risk assessment. Application to bedaquiline and moxifloxacin reproduced clinical QTc observations and demonstrated the value of mechanistic coupling, particularly for characterizing risk under drug–drug interaction scenarios and in patient subgroups with varying physiological characteristics. The sex‐specific differences in repolarization sensitivity captured in the population simulations are consistent with known clinical patterns and illustrate the additional interpretive depth that mechanistic electrophysiology models provide compared to simple concentration‐effect relationships.

The TB disease model provides the biological endpoint where exposure from PBPK, safety signals from the cardiac module, and the pharmacodynamic properties of each drug converge to produce predictions of treatment outcome at the level of bacterial killing and sterilization. Verification against the approximately 50 scenarios reported in the reference Fors et al. publication and its supplementary material confirms that the MoBi reimplementation faithfully captures the core dynamics of TB infection and treatment response. The modular architecture ensures that new drug submodules can be incorporated and existing modules refined without rebuilding the biological core, supporting iterative improvement as new experimental data become available.

Taken together, these four components form a decision framework qualitatively distinct from a collection of individual models. Each element addresses a specific analytical layer of the pharmacological question, and their integration enables a connected analysis from molecular properties through systemic pharmacokinetics, tissue exposure, bacterial dynamics, and cardiac risk within a single reproducible workflow. This connectivity supports a more complete and honest representation of the uncertainty and variability inherent in TB pharmacology than any single modeling layer can provide independently, and creates a platform for systematic analysis of the factors that determine whether a given regimen will achieve the required biological effect in a defined patient population. The framework's open‐source foundation reflects a deliberate scientific and strategic choice. The greatest global burden of TB falls in settings where access to commercial software platforms is constrained and where local modeling capacity is still developing. Making the entire framework available as inspectable, documented, and extendable code through public repositories creates a practical resource for researchers, developers, and regulators working in those settings. It also supports the broader goal of transparency in quantitative drug development, enabling those who use or rely on modeling‐based analyses to examine and challenge the assumptions underlying any prediction or recommendation.

Future development will focus on expanding the mechanistic PBPK library to include additional compounds, refining disease model parameters to improve quantitative agreement with early bactericidal activity data, integrating resistance‐evolution dynamics, and coupling the framework more tightly to population‐simulation tools for patient‐level variability analysis. These developments will progressively expand the practical scope of the framework and its utility across the range of quantitative questions arising in TB drug development, from early candidate selection through dose optimization to regulatory evaluation.

## Funding

This research received funding from the Gates Foundation under Grant INV‐089180.

## Conflicts of Interest

All authors are employees of ESQlabs GmbH.

## Supporting information


**Figure S1:** Workflow of the high‐throughput PBPK framework for in vivo exposure prediction. The pipeline integrates physicochemical inputs derived from experimental measurements or QSAR predictions with Open Systems Pharmacology‐based PBPK model templates to automate compound parameterization, batch simulation, and output analysis across multiple compounds, species, and dosing regimens. In this study, this output is compared to the in vivo‐derived PK profiles of the tuberculosis studies.
**Figure S2:** Relationship between compound properties and model performance. Relationship between lipophilicity (LogD) and fraction unbound per compound, colored by mean log2 AUC fold‐error. Relationship between aqueous solubility (logS) and plasma clearance per compound.
**Table S1:** Inputs and predicted outputs of the high‐throughput PBPK (HT‐PBPK) framework. Compound‐specific inputs are drawn from curated experimental databases when measured values are available and are otherwise predicted from molecular structure using QSAR tools. System (physiology) and study‐design inputs define the simulation context; distribution and elimination are derived from the compound physicochemistry and available clearance information. The framework returns concentration‐time profiles from which exposure metrics are computed.
