# Supplementary material for: Application of an Open‐Source Modular Framework for Pharmacokinetic, Pharmacodynamic, and Safety Simulations to Anti‐Tuberculosis Drugs
Source: CPT Pharmacometrics Syst Pharmacol. 2026 Oct 6;15(10):e70347. doi: 10.1002/psp4.70347 (PMC13639711; doi:10.1002/psp4.70347)
Supplement: Supplementary file 1 [file PSP4-15-e70347-s001.docx]

Supplementary Materials – Application of an open-source modular framework for pharmacokinetic, pharmacodynamic, and safety simulations to anti-tuberculosis drugs

Marco Siccardi^1-2^, Nina Nauwelaerts^1^, Raphaëlle Lesage^1^, Diane Lefaudeux^1^, Vanessa Baier^1^, Venetia Karamitsou^1^, Cleo Demeester^1^, Miao-Chan Huang^1^, Alexander Kulesza^1^ and Stephan Schaller^1^

1- ESQlabs GmbH
2- University of Liverpool

**Figure S1.** Workflow of the high-throughput PBPK framework for *in vivo* exposure prediction. The pipeline integrates physicochemical inputs derived from experimental measurements or QSAR predictions with Open Systems Pharmacology-based PBPK model templates to automate compound parameterization, batch simulation, and output analysis across multiple compounds, species, and dosing regimens. In this study, this output is compared to the *in vivo*-derived PK profiles of the tuberculosis studies.

**Figure S2.** Relationship between compound properties and model performance. Relationship between lipophilicity (LogD) and fraction unbound per compound, coloured by mean log2 AUC fold-error. Relationship between aqueous solubility (logS) and plasma clearance per compound.

**Table S1. Inputs and predicted outputs of the high-throughput PBPK (HT-PBPK) framework.** Compound-specific inputs are drawn from curated experimental databases when measured values are available and are otherwise predicted from molecular structure using QSAR tools. System (physiology) and study-design inputs define the simulation context; distribution and elimination are derived from the compound physicochemistry and available clearance information. The framework returns concentration-time profiles from which exposure metrics are computed.

| **Category** | **Parameter** | **Source / notes** |
| --- | --- | --- |
| Compound physicochemical inputs | Molecular weight | Structure / database |
|  | Lipophilicity (logP / logD) | Experimental or QSAR-predicted |
|  | Acid-base character (pKa, compound type) | Experimental or QSAR-predicted |
|  | Aqueous solubility | Experimental or QSAR-predicted |
|  | Intestinal permeability | Experimental or QSAR-predicted |
|  | Fraction unbound in plasma (fu) | Experimental or QSAR-predicted |
|  | Blood-to-plasma ratio | Experimental where available |
| System (physiology) inputs | Organ compartments, blood flows, cardiac output, tissue composition | Standard OSP/PK-Sim species and population templates |
| Study-design inputs | Route, dose, formulation, dosing schedule, species | Literature-based study designs |
| Derived model parameters | Tissue-to-plasma partition coefficients | Calculated from physicochemistry (standard OSP distribution model) |
|  | Systemic clearance | From available clearance data, or predicted where absent |
| Predicted outputs | Plasma and tissue concentration-time profiles | Model prediction |
|  | AUC, Cmax, Tmax | Derived from concentration-time profiles |
|  | Predicted-to-observed ratio and fold error | Performance metrics vs reference data (when available) |
